# School satisfaction, socioeconomic status, and life satisfaction: testing the mediating role of perceived future employability and the moderating role of gender

**DOI:** 10.3389/fpsyg.2026.1779829

**Published:** 2026-05-13

**Authors:** Yavuz Aslan, Orhan Koçak, Abdulhalim Çelik, Abdulmohsen Mohammed Abdullah Alkhulayfi, Juan Gómez-Salgado, Murat Yıldırım

**Affiliations:** 1 Department of Social Work, Mardin Artuklu University, Mardin, Türkiye; 2 Department of Social Work, İstanbul University-Cerrahpasa, İstanbul, Türkiye; 3 Industrial Relations & Labor, Kocaeli University, Körfez, Türkiye; 4 Department of Business Administration, Faculty of Economics and Administration, King Abdulaziz University, Jeddah, Saudi Arabia; 5 Department of Sociology, Social Work and Public Health, Faculty of Labour Sciences, University of Huelva, Huelva, Spain; 6 Safety and Health Postgraduate Program, Universidad Espíritu Santo, Guayaquil, Ecuador; 7 Department of Psychology, Faculty of Science and Letters, Ağrı İbrahim Çeçen University, Ağrı, Türkiye; 8 Psychology Research Centre, Khazar University, Baku, Azerbaijan

**Keywords:** life satisfaction, perceived future employability, school satisfaction, socioeconomic status, Turkish university students

## Abstract

This study examines the mediating role of perceived future employability and the moderating role of gender in the relationship between school satisfaction, socioeconomic status, and life satisfaction. Drawing on both psychological perspectives and Pierre Bourdieu’s theory of capital and habitus, perceived future employability is conceptualized as a socially embedded construct linking structural conditions to individual well-being. A total of 2,400 university students (mean age = 20.89 ± 2.32; 1,648 females) participated in the study, which was conducted via online platforms and used a correlational research design. The findings indicated that school satisfaction was positively associated with both perceived future employability and life satisfaction, whereas socioeconomic status was negatively associated with perceived future employability but positively associated with life satisfaction. Perceived future employability was positively associated with life satisfaction and had a mediating role in the relationships of school satisfaction and socioeconomic status with life satisfaction (school satisfaction: *γ* = 0.039, 95% CI [0.023, 0.056]; socioeconomic status: *γ* = −0.015, 95% CI [−0.028, −0.006]). In addition, gender was found to moderate the relationships between school satisfaction, socioeconomic status, and perceived future employability. Moderation results indicated that as school satisfaction increased, perceived future employability increased more strongly among females than males, whereas as socioeconomic status increased, perceived future employability decreased more among females than males. The results offer important implications for educational institutions and policymakers seeking to increase students’ overall life satisfaction and future career opportunities. Promoting supportive educational environments and addressing socioeconomic and gender-based inequalities in education and employment can help institutions positively influence students’ satisfaction and perceptions of future employment opportunities.

## Introduction

The quality of education at universities is closely associated with students’ satisfaction with their educational experiences, which, in turn, relates to important outcomes such as academic persistence and graduation rates. Students’ satisfaction is shaped by the extent to which educational services meet their expectations and by institutional characteristics such as academic resources, administrative structures, campus life, and support services (Ulusoy et al., 2020; Gatfield et al., 1999). In this context, students’ sense of belonging and the guidance they receive regarding their future careers also play a crucial role in shaping their satisfaction with higher education.

Student satisfaction has been consistently linked to broader indicators of well-being, particularly life satisfaction. As a cognitive evaluation of one’s overall life conditions, life satisfaction encompasses multiple life domains, including education (Tian et al., 2014). Accordingly, students’ satisfaction with their educational experiences is an important contributor to their overall life satisfaction. At the same time, the perceived quality of education is closely connected to students’ expectations regarding their future employment opportunities. In contemporary labor markets characterized by rapid technological change and increasing competition, higher education is expected to equip individuals with the skills and competencies required for successful participation (Butt and Rehman, 2010; Asonitou, 2015). Therefore, students’ perceptions of their future employability have become a critical outcome of educational experiences.

However, perceptions of employability cannot be fully understood through individual competencies and educational experiences alone. These perceptions are also shaped by broader social and structural conditions. In this regard, Pierre Bourdieu’s theory of capital and habitus provides an important framework for understanding how individuals’ expectations about their future careers are socially structured (Bourdieu, 1986, 1990). From a Bourdieusian perspective, socioeconomic status can be conceptualized as a proxy for individuals’ access to economic and cultural capital, which shapes their habitus—that is, their internalized dispositions, expectations, and perceptions of future opportunities. Within this framework, perceived future employability reflects how individuals interpret their position within the social structure and evaluate their future career prospects. Therefore, perceived future employability can be understood as a mechanism through which structural inequalities associated with socioeconomic status are internalized and translated into subjective well-being outcomes such as life satisfaction.

Although previous studies have examined the relationships among school satisfaction, perceived employability, and life satisfaction, these relationships have largely been addressed within variable-centered and psychologically oriented frameworks (Gunawan et al., 2021). Such approaches tend to conceptualize perceived employability primarily as an individual-level cognitive construct, overlooking how it is shaped by structural conditions. Moreover, while mediation models involving employability have been explored in prior research, the role of perceived future employability as a socially structured mechanism linking socioeconomic status to life satisfaction remains insufficiently examined.

Against this background, the present study extends the existing literature by integrating psychological and sociological perspectives. Specifically, it conceptualizes perceived future employability not merely as a mediator, but as a socially embedded mechanism through which structural conditions, particularly socioeconomic status, are translated into individual well-being outcomes. Therefore, this study aims to examine the relationships among school satisfaction, socioeconomic status, perceived future employability, and life satisfaction among university students, and to test the mediating role of perceived future employability and the moderating role of gender in these relationships.

### School satisfaction

One important approach to explaining the relationship between education and employment is the ‘Human Capital Theory.’ (Öztürk, 2005). According to the theory, investments in individuals shorten the development process (Schultz, 1971). As investments are made in people, both national income and personal gains increase. Among these investments, education has an important place. According to human capital theory, the knowledge and skills that individuals acquire through education and work experience increase their productivity in working life and lead to higher wages (Becker, 2009). Accordingly, investments in education should be made to increase labor force productivity.

It has been stated that individuals with higher levels of education have certain advantages, such as earning higher incomes and being more likely to be employed than those with lower levels of education (Mincer, 1993). The most important reason is that education increases productivity by equipping individuals with new knowledge and skills. This shows an important relationship between education and employment. Accordingly, assuming that education plays an important role in employment, as the quality of education increases, the advantages of employment and high income will become more apparent. Thus, individuals’ satisfaction will also be positively affected.

Satisfaction is the feeling of happiness that individuals feel after their needs, desires, and expectations are met (Zemke, 2000). On the other hand, student satisfaction is the attitude formed after evaluating students’ experiences with their education. Student satisfaction usually occurs in universities after meeting student needs (Elliott and Healy, 2001). Since universities are also a service sector, they should consider students’ needs and expectations (Cheong Cheng and Ming Tam, 1997). Therefore, student satisfaction is decisive in measuring university education and training activities (El Ansari, 2002). In this context, many studies have been conducted to measure the satisfaction of students in universities (Chuah and Sri Ramalu, 2011; Sharma and Sharma, 2018).

School satisfaction refers to students’ subjective cognitive evaluations of the quality of their lives at school (Baker and Maupin, 2009). In recent years, many studies have examined the effects of students’ school satisfaction on their achievement (Archibald, 2006; Dincer and Uysal, 2010; Stewart, 2008). Studies show that school environments with desired characteristics positively affect student achievement (Egelioğlu et al., 2011; Hoy and Hannum, 1997). However, the quality of the education students receive also plays an important role in this success. Therefore, a qualified education and a school environment that meets students’ wishes and expectations affect students’ satisfaction with school.

School satisfaction is an important factor affecting students’ future professional careers. Studies have found a significant relationship between students’ academic satisfaction and professional expectations (Koçak et al., 2021; Yu and Yang, 2013). Students’ occupational expectations are generally to have a job suitable for the quality of the education received after graduation (Kuzgun, 2009). Therefore, the perception that they can work in a job they want after graduation is closely related to students’ school satisfaction. From a sociological perspective, educational environments may reinforce or challenge individuals’ perceptions of their future positions within the social structure. Students who experience higher levels of school satisfaction may develop stronger confidence in their future employability, as positive educational experiences can enhance both perceived competence and social positioning. Therefore, the following hypothesis was formulated.

*H1*: School satisfaction is associated with perceived future employability.

### Life satisfaction

Subjective well-being, discussed in positive psychology, is a state of well-being that emerges as a result of individuals’ subjective evaluations of their lives (Diener and Ryan, 2009). In addition, subjective well-being, a psychological factor, is related to the individual’s life satisfaction (Lucas and Diener, 2009). *Life satisfaction* is a cognitive and judgmental evaluation that compares individuals’ criteria with their expectations (Haybron, 2004). In other words, life satisfaction is the cognitive judgment of individuals towards all areas of life, such as family, school, work, and social environment (Diener et al., 1985). When the literature is examined, it is understood that life satisfaction is related to an individual’s self-esteem (Hawi and Samaha, 2016), life goals (Olčar et al., 2019), vocational expectations (Aslan and Koçak, 2023), academic achievement (Klapp et al., 2023), and school satisfaction (Koçak et al., 2021). School satisfaction is related to the quality of education students receive, the tools and equipment they use at school, and academic activities. The quality of the education students receive in schools, and the quality of the relationships they establish with others, also affect their lives. Studies have shown that school satisfaction significantly affects students’ success, attendance, and psychological well-being (DeSantis King et al., 2006; Verkuyten and Thijs, 2002). Beyond individual experiences, life satisfaction can also be understood as being shaped by individuals’ positions within broader social structures. Educational experiences and future expectations may influence how individuals evaluate their life conditions within these structures. Therefore, the following hypothesis was developed.

*H2*: School satisfaction is associated with life satisfaction.

In addition, employability also significantly affects an individual’s psychological well-being. When the studies were examined, a positive and significant relationship was found between perceived future employability and psychological well-being (Alkın et al., 2020; Kinnunen et al., 2011). In the study conducted by Vanhercke et al. (2015), individuals with a high perception of future employability have control over their lives, which positively affects their psychological well-being. In addition, employability also affects life satisfaction, an important component of psychological well-being. Studies have found a significant positive relationship between employability and life satisfaction (Alkın et al., 2020; De Cuyper et al., 2008; Praskova et al., 2015). Therefore, the following hypothesis was developed.

*H3*: Perceived future employability is associated with life satisfaction.

### Socioeconomic status

Socioeconomic status (SES) refers to an individual’s social position regarding income, occupation, and social class (Sarsani, 2011). Important indicators of socioeconomic status are income, education, and occupation. There is no universally accepted definition of socioeconomic status. High socioeconomic status refers to high-status occupation, high income, and high living standards; low socioeconomic status refers to low-status occupation, low income, and poor living conditions (Sarsani, 2011). Studies have revealed that socioeconomic indicators are important determinants of an individual’s health (Nouraei Motlagh et al., 2022), and quality of life (Razzaque et al., 2010). In general, the higher the individual’s socioeconomic status, the higher the level of life satisfaction. However, objective socioeconomic status indicators, such as increasing income and occupation, lose their effect on life satisfaction (Horanicova et al., 2022). On the contrary, subjective socioeconomic status, which compares the individual with the group he/she is in, has become a more significant and positive determinant of life satisfaction (Anderson et al., 2012).

In addition to affecting life satisfaction, socioeconomic indicators also affect employability. Studies have been conducted on which factors affect employability. (Sparrow and Wu, 1998) found that cultural values impact employability. Harry et al. (2018) found that low socioeconomic status affects employability. Another study found that factors such as personality and gender are determinants of employability (Qureshi et al., 2016). From a sociological perspective, SES is not only a material condition but also reflects access to different forms of capital and shapes individuals’ expectations, aspirations, and perceived opportunities. These structural conditions may influence how individuals evaluate their future employability. Based on the literature, the following hypotheses were developed.

*H4*: SES is positively associated with perceived future employability.

*H5*: SES is positively associated with life satisfaction.

### Perceived future employability as a mediator

School satisfaction and socioeconomic status can affect students’ perceived future employability, which in turn can affect their happiness, well-being, and life satisfaction. *Perceived employability* is defined as an individual’s self-sufficiency by revealing his/her potential in the labor market (Hillage and Pollard, 1999). While perceived employability focuses on an individual’s current employability, perceived future employability describes an individual’s professional self at some point in the future (Gunawan et al., 2021). For university students, perceived future employability is an assessment of their personal characteristics, skills, experiences, networks, and labor market knowledge after they finish their education and feel ready to work.

However, perceived future employability can be understood not only as an individual cognitive evaluation but also as a socially constructed perception shaped by broader structural conditions. From a sociological perspective, Pierre Bourdieu’s theory of capital and habitus suggests that individuals’ future expectations and career perceptions are deeply influenced by their social background and access to various forms of capital (Bourdieu, 1986, 1990). In this context, perceived future employability may serve as a mechanism by which structural factors, such as socioeconomic status and educational experiences, are internalized and reflected in individuals’ subjective evaluations of their lives. Therefore, rather than a purely psychological construct, perceived future employability can be conceptualized as a mediating process linking structural conditions to life satisfaction.

Studies have been conducted on the effect of school satisfaction (James and Yun, 2018; Sulistiobudi and Wijayanti, 2019), and socioeconomic status (Sparrow and Wu, 1998) on university students’ perceived future employability. In addition, studies have also been conducted to investigate whether the perceived future employability has an impact on students’ well-being and life satisfaction (Güneş and Acar, 2022; Özçelik Bozkurt and Gökhan Özkoç, 2019). In contrast to studies on students’ school satisfaction, socioeconomic status, well-being, and life satisfaction, very few studies have examined the role of perceived future employability in students’ lives. Therefore, this study analyzed the mediating effect of perceived future employability on students’ school satisfaction and the effect of socioeconomic status on their life satisfaction. Therefore, the following hypotheses were hypothesized.

*H6*: Perceived future employability has a mediating role in the relationship between school satisfaction and life satisfaction.

*H7*: Perceived future employability has a mediating role in the relationship between socioeconomic status and life satisfaction.

### Gender as a moderator

Gender differences affect students’ school satisfaction and employability. Shaped by biological, psychological, and social factors, gender differences manifest themselves in areas such as abilities, interests, social relationships, self-concept, and depression (Perry and Pauletti, 2011). Research reveals how gender differences affect the way people experience different aspects of life. For example, boys reported higher satisfaction than girls in appearance, self-confidence, and leisure time (Chen et al., 2020; Verkuyten and Thijs, 2002).

One of the areas where gender differences are most evident is in school. Many studies have shown that girls and boys have different experiences in school. Studies have shown that male students have low academic achievement and do not like school (Buluş Kirikkaya, 2011; Halpern, 1997). However, it is understood that girls have higher school satisfaction levels than boys (Chen et al., 2020).

Although school satisfaction is higher for girls than for boys, employability remains lower. Studies show that men have better options in the labor market and are more easily employed than women (McQuaid and Lindsay, 2005). In addition to gender, socioeconomic factors also affect employability. Mishra et al. (2017) revealed the effects of socioeconomic conditions on employability with their study. In addition, (Cifre et al., 2018) revealed the impact of socioeconomic status and factors such as gender on employment in their study in India. Based on the literature, the following hypotheses were developed.

*H8*: Gender has a moderating role in the relationship between school satisfaction and perceived future employability.

*H9*: Gender moderates the relationship between socioeconomic status and perceived future employability.

### Present study

**Figure 1:**
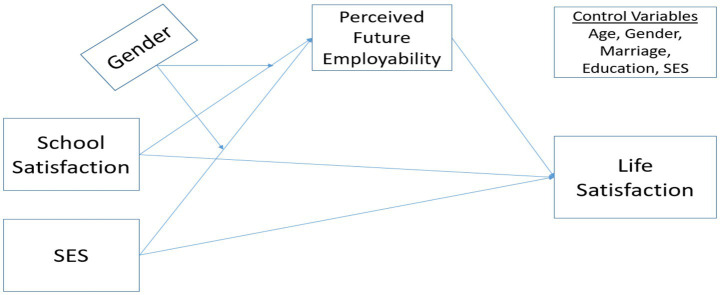
Concept model of the research.

This study examines the mediating role of perceived future employability and the moderating role of gender in the relationship between school satisfaction, socioeconomic status, and life satisfaction. School satisfaction, a subjective evaluation of students’ experiences at school, is affected by many life-related variables. School satisfaction is related to the education received, the tools and equipment used, academic achievement, family, friends, and the environment in which they live. In addition, school satisfaction is also related to students’ professional careers. A quality education and a school environment that meets expectations affect academic achievement. Academic success, in turn, affects expectations for future employment based on the quality of education received after graduation. The perceived future employability affects individuals’ psychological well-being and, thus, their life satisfaction. This study assumes that school satisfaction affects students’ perceived future employability, and that perceived future employability, in turn, affects their life satisfaction. In addition, socioeconomic status affects students’ perceived future employability, which in turn affects their life satisfaction. For this purpose, the conceptual model shown in Figure 1 was designed. Direct, indirect, and moderation analyses were used in the conceptual model. Hypotheses were defined and tested for each relationship. These relationships were hypothesized to vary by gender.

## Methods

### Participants and process

The research was conducted online using the SurveyMonkey platform. A total of 2,400 individuals participated in the survey, which was conducted only among university students. Of the participants, 1,648 were women, 752 were men, and the mean age was 20.89 (SD = 2.32). The participants were recruited from public universities in Türkiye, thereby providing a relatively homogeneous institutional context with respect to educational structure and access to resources. The study was conducted within a single-country context, although the research team consists of international scholars; this design was preferred to ensure contextual consistency when examining variables such as socioeconomic status, perceived employability, and gender, which are sensitive to cultural and institutional differences. After obtaining ethics committee approval for the research, the survey was distributed to academics and university students across different universities. In the survey, participants’ consent was obtained before they began answering the questions. Ultimately, 2,530 university students participated in and supported the research, but because some lacked data, the responses of 2,400 participants were evaluated. Participants could leave the survey at any time. The data does not contain any participant’s private information. The average time to complete the survey was 8–9 min. The research was carried out in accordance with the Declaration of Helsinki and with approval from the ethics committee of Kocaeli University.

### Measures

Demographic variables were included in the survey to characterize the participants, including gender, age, parental education, and family income. Socioeconomic status (SES) was operationalized using three indicators: maternal education level, paternal education level, and family income, each measured on a five-point scale. To ensure comparability across indicators, all variables were first standardized using z-scores. A composite SES index was then calculated by averaging these standardized scores, assigning equal weight to each component, as they are considered complementary indicators of socioeconomic position. This approach is consistent with previous research in which SES has been constructed by combining parental education and family income (Aarø et al., 2009; Louis and Zhao, 2016; Wickrama et al., 2009).

School Satisfaction Scale was developed by (Demirli and Kerimgil, 2009). The scale was developed using the scanning model. During the development process, the creation of the items pool, the preparation of the trial scale, the application of the scale, the finding of the item-total correlations, and the calculation of the internal consistency were provided, respectively. The scale consists of 30 items and is rated on a five-point Likert scale ranging from 1 (strongly disagree) to 5 (strongly agree). In the scale, there are items such as “I am studying in a department related to the profession I want”. The higher the total score obtained from the scale, the higher the student’s satisfaction with the university. The KMO value of the scale was 0.94, the Bartlett test was 8321.183 (*p* < 0.05), and the Cronbach Alpha reliability coefficient of the scale was 0.92. The Cronbach Alpha of the current study was 0.945.

The Perceived Future Employability Scale (PFES) was developed by (Gunawan et al., 2018) to assess individuals’ perceptions of their future employment prospects. The scale was adapted into Turkish by Akın et al. (2020). The scale consists of 24 items and is rated on a six-point Likert scale ranging from 1 (strongly disagree) to 6 (strongly agree). A sample item is “I will gain the knowledge I need to get the job I want.” Higher scores indicate higher levels of perceived future employability. In the current study, the internal consistency of the scale was acceptable (Cronbach’s *α* = 0.878).

Satisfaction with Life Scale (SWLS) was developed by Diener et al. (1985). It was adapted into Turkish by (Dağlı and Baysal, 2016). The Satisfaction with Life Scale consists of 5 items to assess individuals’ life satisfaction. The scale is a five-point Likert type. According to this, it was graded as totally agree (5), agree (4), undecided (3), disagree (2), and strongly disagree (1). The internal consistency (Cronbach’s alpha) of the Turkish version of the Satisfaction with Life Scale was 0.88. Among the scale items are statements such as “In most ways my life is close to my ideal.” An increase in the scale score indicates that individuals’ life satisfaction will be higher. The Cronbach’s alpha obtained in this study was 0.86.

### Common method bias

To reduce the potential risk of common method bias, several procedural remedies were implemented during data collection. Participants were informed that their responses would remain anonymous and confidential, participation was voluntary, and there were no right or wrong answers, thereby reducing evaluation apprehension and social desirability bias. In addition, common method bias was statistically assessed using Harman’s single-factor test. The results indicated that a single factor accounted for less than 50% of the total variance, suggesting that common method bias was not likely to be a serious concern in this study.

### Data analyses

After the data was collected, it was imported into MS Excel, and unnecessary entries were deleted. Afterward, the database was imported into SPSS. Confirmatory factor analysis was performed using IBM AMOS to test the construct validity of the measurement model depicted in Figure 1. After reporting the measurement model’s construct validity and fit indices, factor scores were estimated in AMOS using the validated measurement model and then exported to SPSS for subsequent analyses. These factor scores were then exported and used as observed variables in SPSS PROCESS Macro for moderation analyses. Correlation, reliability, skewness, and kurtosis were analyzed with the newly created data file. After providing the fit values for the measurement model, total, direct, and indirect effects were tested using path analysis, which is suitable for the model. SPSS Process Macro Plug-in and simple slope were used for moderation analysis. Analyses were performed with a 95% confidence interval and 5,000 bootstraps. Given the unequal gender distribution in the sample, gender was explicitly included as a moderator in the analyses, and moderation effects were examined using the PROCESS Macro to account for potential group differences.

## Results

### Preliminary analysis

To test the measurement model developed in the study, confirmatory factor analysis was conducted using IBM AMOS 24. As a result of the first analysis, it was understood that the required fit values were slightly below expectations. The suggested reasonable number of covariances in the proposed modification indices was used to provide the desired fit values. After the performed covariances, it was determined that the model fit values matched the cut-off criteria determined by Kline (GFI, TLI, NFI, IFI, CFI, and CFI > 0.90, CMIN /df < 5, RMSEA <0.08) (Kline, 2016). Thus, the final fit values of the measurement model were found to be acceptable when compared to Kline’s cut-off values, as shown in Table 1.

**Table 1:** Confirmatory factor analysis results.

Measure	Estimate	Threshold	Interpretation
CMIN/DF	4.549	Between 1 and 3	Acceptable
GFI	0.950	>0.95	Excellent
NFI	0.952	>0.95	Excellent
TLI	0.957	>0.95	Excellent
CFI	0.962	>0.95	Excellent
SRMR	0.048	<0.08	Excellent
RMSEA	0.038	<0.08	Excellent

Based on the measurement model fit indices from the study’s confirmatory factor analysis, a new data file was created using imputation. Analyses were conducted to assess correlations, means, skewness, and kurtosis for the new variables, as shown in Table 2. It was observed that the skewness and kurtosis values for the variables’ normality were within expected ranges. As a result of the correlation analysis, positive relationships were observed between school satisfaction and socio-economic status (B = 0.071, *p* < 0.01), perceived future employability (B = 0.371, *p* < 0.01), and life satisfaction (B = 0.450, *p* < 0.01). Socio-economic status was found to have a negative association with perceived future employability (B = −0.075, *p* < 0.01) and a positive association with life satisfaction (B = 0.297, *p* < 0.01). A positive correlation was observed between perceived future employability and life satisfaction (B = 0.227, p < 0.01).

**Table 2:** Correlations, means, std. deviations, Skewness, Kurtosis.

No.	Variables	1	2	3	4
1	School satisfaction	1			
2	Socio-economic status (SES)	0.071**	1		
3	Perceived future employability	0.371**	−0.075**	1	
4	Life satisfaction	0.450**	0.297**	0.227**	1
	Mean	3.081	0.977	3.930	2.294
	Standard deviation	0.645	0.377	0.924	0.725
	Skewness	−0.400	0.678	−0.881	0.652
	Kurtosis	0.475	0.144	0.157	−0.377

**p < 0.01.

### Direct and indirect path analyses using SEM

After the fit indices of the measurement model met Kline’s cutoff values, path analyses were conducted to examine direct and indirect effects. In the path analysis conducted in accordance with the conceptual model, it was determined that school satisfaction increased perceived future employability and life satisfaction, whereas socio-economic status decreased perceived future employability but increased life satisfaction. In addition, it was understood that perceived future employability, the mediator variable, significantly increased life satisfaction. The indirect analysis determined that perceived future employability mediated the effect of school satisfaction (*γ* = 0.039, *p* = 0.001, 95% CI [0.023, 0.056]) and socio-economic status (γ = −0.015, *p* = 0.003, 95% CI [−0.028, −0.006]) on life satisfaction. These indirect effects can be considered small in magnitude, indicating that while statistically significant, the mediation effects are modest in practical terms. According to the mediation data, the upper and lower confidence intervals did not include zero, as shown in Table 3.

**Table 3:** Direct and indirect SEM analyses.

Direct paths	Unst. Est.	S.E.	C:R.	*p*
School satisfaction → PF employability	0.46	0.034	13,58	***
SES → PF employability	−0.174	0,052	−3,322	***
School satisfaction → LS	0.384	0.029	13,202	***
SES → LS	0.398	0.048	8,244	***
PF employability → LS	0.085	0.018	4,624	***

Indirect paths	Unst. Est.	Lower	Upper	*p*-value	Std. Est.	
School satisfaction → PF employability → LS	0.039	0.023	0.056	0.001	0.036	***
SES → PF employability → LS	−0.015	−0.028	−0.006	0.003	−0.009	**

PF employability, perceived future employability; SES, socio economic status; LS, life satisfaction.

### Moderator analyses

**Figure 2:**
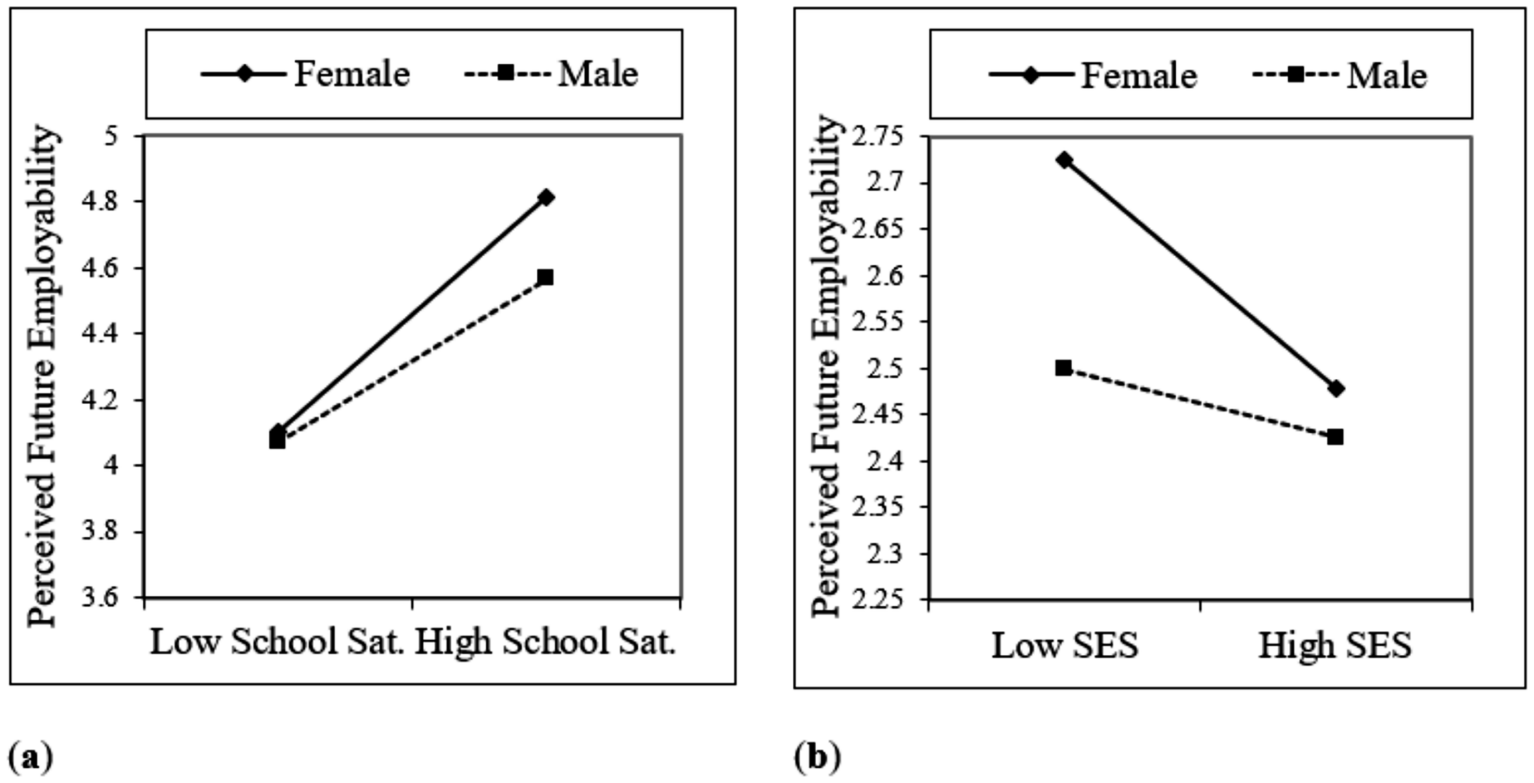
Moderation effects: **(a)** The moderating role of gender in the relationship between school satisfaction and perceived future employability; **(b)** The moderating role of gender in the relationship between socioeconomic status and perceived future employability.

In the current study, a moderator analysis was conducted between the independent and mediator variables, using gender as the moderator. For this analysis, the interaction variables generated (School Satisfaction × Gender; SES × Gender) and Process Macro Model 7 were used. The moderator analysis using interaction variables indicated that both variables had a statistically significant effect. It was understood that gender moderates the relationships between school satisfaction (B = −0.1831, p < 0.01) and socioeconomic status (B = 0.2145, *p* < 0.05) and perceived future employability. Accordingly, in Figure 2a, as school satisfaction increased, future employability increased more among females than among males, suggesting that female students may be more responsive to educational experiences when forming expectations about their future careers. However, as socio-economic status increased, perceived future employability decreased more among females than among males, as shown in Figure 2b, which may indicate that female students with higher socioeconomic backgrounds develop higher expectations and greater sensitivity to labor market competition, leading to more critical evaluations of their future employability.

## Discussion

School satisfaction can vary greatly from person to person. It often depends on various factors such as the quality of education, teaching methods, relationships with teachers and peers, the availability of resources, extracurricular activities, and the overall environment. Schools need to focus on academic success and fostering an environment where students feel valued, supported, and encouraged to explore their interests and passions. When students feel satisfied with their school experience, it can positively impact their academic performance, mental health, and overall well-being. Academic satisfaction can significantly impact perceived future employability. When students are satisfied with their academic experience, it often translates into the acquisition of relevant skills, knowledge, and competencies that are highly valued in the job market.

This study examined the role of perceived future employability as a mediator in the relationships between school satisfaction and life satisfaction, and between socioeconomic status and life satisfaction. Moreover, it examined the potential moderating effect of gender on the associations among school satisfaction, socioeconomic status, and perceived future employability. Findings from correlation, mediation, and moderation analyses supported hypotheses H1–H9. Subsequent sections detail the assessments of direct, mediation, and moderation analyses by established hypotheses and existing literature outcomes.

### Direct effects

The findings indicated a significant positive association between school satisfaction and perceived future employability. Higher levels of school satisfaction were associated with greater perceived future employability. Although the direct relationship between school satisfaction and perceived employability has received limited attention in the literature, previous studies have linked perceptions of employability to factors such as academic performance and student engagement (Pan and Lee, 2011; Ma and Bennett, 2021). In this context, school satisfaction may be associated with perceived employability through its relationship with students’ academic experiences and engagement. A positive educational environment may be linked to the development of key competencies such as critical thinking, problem-solving, communication, and collaboration, which are valued in the labor market. In addition, supportive school contexts may provide opportunities such as internships, practical experiences, and mentoring that strengthen students’ perceptions of their future employability. From a Bourdieusian perspective, this finding may reflect the alignment between individuals’ habitus and the educational field, suggesting that access to resources and compatibility with institutional expectations may shape how students evaluate their future career prospects.

The findings indicated a significant positive association between school satisfaction and life satisfaction. This result is consistent with previous studies demonstrating a positive link between school satisfaction and life satisfaction (Gempp and González-Carrasco, 2021; Gül and Koşan, 2022). Students’ contentment with their educational environment intertwines with their psychological well-being, social dynamics, and personal growth. A favorable scholastic experience significantly contributes to the psychological welfare of adolescents and young adults by fostering improved emotional equilibrium, reducing stress, and cultivating a positive mental disposition. Furthermore, fostering positive social connections within the school milieu plays a pivotal role in fortifying enduring social bonds, thereby augmenting overall life satisfaction. From a Bourdieusian perspective, this finding may reflect the alignment between individuals’ habitus and the norms of the educational field, suggesting that students who are better attuned to the expectations of their educational environment may report higher levels of well-being and life satisfaction.

The findings indicated a significant positive association between perceived future employability and life satisfaction. Higher levels of perceived future employability were associated with greater life satisfaction. This result is consistent with previous studies reporting a positive relationship between perceptions of employability and life satisfaction (Alkın et al., 2020; Praskova et al., 2015). Perceived future employability significantly shapes overall life satisfaction by serving as a psychological anchor, positively influencing career-oriented plans, mitigating financial concerns, and nurturing self-assurance. Maintaining faith in future career prospects engenders optimism, mitigating uncertainty and augmenting one’s overall life perspective. Moreover, it assuages financial apprehensions, fostering a sense of security and bolstering life satisfaction. Additionally, this perception elevates self-esteem, fosters a constructive self-identity, and helps individuals lead more gratifying lives. From a Bourdieusian perspective, these findings can be interpreted within Pierre Bourdieu’s framework of habitus and capital, suggesting that individuals’ perceptions of future employability reflect their internalized sense of opportunities and constraints shaped by their social background, which in turn influences their overall life evaluations.

Empirical analysis revealed a significant relationship between socioeconomic status (SES) and perceived future employability. Although a positive association was theoretically expected, the findings indicated a significant negative relationship. From a theoretical standpoint, individuals with higher SES are generally assumed to have greater access to economic, cultural, and social resources, which should enhance their confidence in future employment opportunities. However, the present findings point to an alternative interpretation. It is possible that individuals from higher socioeconomic backgrounds develop elevated expectations and greater awareness of labor market competition, which may lead to more critical self-evaluations and, consequently, lower perceived employability. In contrast, individuals from lower SES backgrounds may display stronger motivation, resilience, and adaptive expectations, which could support more positive perceptions of future employability despite structural disadvantages. While previous studies have highlighted the role of socioeconomic conditions in shaping employability (Harry et al., 2018; Sparrow and Wu, 1998), the current findings suggest that this relationship may not always operate in a straightforward positive direction. From a Bourdieusian perspective, these results can be interpreted within Pierre Bourdieu’s framework of capital, indicating that differences in access to economic, cultural, and social resources shape how individuals interpret their future opportunities and employability.

A significant positive association was found between socioeconomic status and life satisfaction. Higher socioeconomic status was associated with greater life satisfaction. This finding is consistent with previous studies reporting a positive relationship between socioeconomic status and life satisfaction (Anderson et al., 2012; Razzaque et al., 2010). Socioeconomic status significantly influences life satisfaction. Elevated SES correlates with improved access to resources, education, healthcare, and employment opportunities. Individuals with higher SES often experience greater life satisfaction due to improved living standards, financial stability, and access to a range of amenities. From a Bourdieusian perspective, these findings can be understood within Pierre Bourdieu’s framework of capital, where individuals’ access to economic, cultural, and social resources shapes their living conditions and contributes to differences in overall life satisfaction.

### Indirect effects

The mediation condition was fulfilled by perceived future employability, which served as the mediating variable in the study, forming a significant relationship with the independent variables of school satisfaction and socioeconomic status, and with the dependent variable, life satisfaction. Therefore, hypotheses H6 and H7 were confirmed in the study.

School satisfaction indirectly affects life satisfaction through perceived future employability. This suggests that increasing school satisfaction will lead to a greater perceived employability in the future and, as a result, greater life satisfaction. To date, no study has focused on the mediating effect of perceived future employability on the relationship between school satisfaction and life satisfaction. This study contributes to the literature by revealing the mediating role of perceived future employability in the relationship between school satisfaction and life satisfaction.

School satisfaction and life satisfaction are important areas of study. School satisfaction, or how content students are with their educational experience, can impact their overall life satisfaction. Similarly, perceived future employability, or the belief in one’s ability to secure a job in the future based on their education, can significantly influence life satisfaction. Considering these factors, a positive perceived future employability stemming from satisfaction with one’s educational experience could mediate or act as a bridge between school and overall life satisfaction. When students feel satisfied with their education and believe it will lead to better job prospects, this could contribute significantly to their happiness and fulfillment.

Socioeconomic status (SES) indirectly influences life satisfaction through the intermediary factor of perceived future employability. The hypothesis posits that a decline in socioeconomic status is associated with increased perceived future employability, which subsequently leads to increased life satisfaction. Notably, this study makes a novel contribution to the existing literature by exploring the mediating role of perceived future employability. This dimension has yet to be extensively investigated in prior research. While it is anticipated that individuals with high socioeconomic status will exhibit higher perceptions of employability, several factors can help explain the heightened employability perceptions among those with low socioeconomic status. Elements such as formidable competitive conditions, motivation, versatile skill sets, a strong work ethic, educational and professional development, resilience, and adept problem-solving capabilities can effectively enhance the perceived employability of individuals with low socioeconomic status. These individuals often demonstrate heightened resilience and motivation when navigating challenges, making them better prepared and more competitive in the job search process. Furthermore, a focus on diverse skills and abilities can cultivate a favorable impression with employers, thereby strengthening overall employability. Hence, this situation may contribute positively to their overall life satisfaction. From a Bourdieusian perspective, this mediating process can be understood within Pierre Bourdieu’s framework of habitus and capital, where individuals’ perceptions of future employability reflect the internalization of structural conditions such as socioeconomic background and educational experiences, which in turn shape their overall life satisfaction.

### Moderation effects

Moderation analyses were conducted to identify potential differences in the effects of the independent variables—school satisfaction and socioeconomic status—on the mediating factor, perceived future employability, across genders. The outcomes of the moderation analysis conducted using the SPSS Process Macro Model 7 are depicted in Figures 2a,b. Subsequently, Hypotheses 8 (H8) and 9 (H9) found support, emphasizing the noteworthy moderating impact of school satisfaction and socioeconomic status on the relationship between school satisfaction and the perceived future employability. It is established that heightened school satisfaction yields a more considerable enhancement in the perceived future employability among women than men. Conversely, with an increase in socioeconomic status, women tend to experience a comparatively steeper decline in perceived future employability than men.

This insight illuminates a robust relationship between school satisfaction and future employability, particularly accentuated within the female cohort. Extant research indicates that women report higher school satisfaction than men (Chen et al., 2020). Paradoxically, empirical evidence reveals a higher employment rate among men vs. women (McQuaid and Lindsay, 2005). This divergence warrants multifaceted interpretation; factors that augment women’s school satisfaction may substantially influence their career trajectories, underscoring the pivotal role of educational experiences in shaping their professional prospects. Moreover, a plausible link might exist between women’s satisfaction with their education and their subsequent success in professional endeavors. This finding underscores the intricate interplay of educational experiences, gender dynamics, and their disparate outcomes. An in-depth understanding of the relationship between heightened academic satisfaction among women and their perceived future employability is imperative for devising more equitable educational policies and fostering inclusive job opportunities. From a critical gender perspective, these patterns can be further understood through the lens of Harding’s (1991)standpoint theory, which suggests that women’s experiences and perceptions are shaped by their social positioning within gendered power structures. In this context, women’s higher school satisfaction may not translate into equally strong perceptions of employability, as structural inequalities in labor markets may constrain the conversion of their educational experiences into career opportunities.

Conversely, a discernible nexus emerges between men’s socioeconomic status and future employability. Scholarly discourse accentuates that the prevailing work environment influences gender-based perceived future employability (Cifre et al., 2018). Nevertheless, prevailing data suggest that men encounter more diverse employment options and experience greater ease in securing employment than their female counterparts in the labor market (McQuaid and Lindsay, 2005). The intricate interplay among societal gender norms, networking advantages, and perceptions of education and skills could explain this situation. Societal expectations and gender roles potentially reinforce the notion that elevated status confers amplified job opportunities for men.

Additionally, men’s expansive professional networks and heightened communication prowess in business settings may strengthen the association between higher socioeconomic status and future job prospects. Moreover, the perceived correlation between men with elevated socioeconomic standing and their presumed superior education and skills underpins their heightened perception of future employability. This observation necessitates in-depth scrutiny to delineate gender-based perceptual disparities and discern the influences of societal norms, workplace inequities, and perceived opportunities.

## Limitations

In this study, the investigation centered on the mediating role of perceived future employability in the influence of school satisfaction and socioeconomic status on life satisfaction, and examined gender’s moderating effect on the relationships among school satisfaction, socioeconomic status, and perceived future employability. Given the cross-sectional nature of this research methodology, caution is warranted when interpreting the outcomes, given its limited scope for representing broader societal contexts. The study’s reliance on a limited participant pool limits the generalizability of the findings beyond the specific demographics involved. In addition, the use of convenience sampling via an online survey may introduce selection bias, as participation was limited to individuals with access to digital platforms and a willingness to respond, which may affect the representativeness of the sample. Furthermore, the exclusive use of online quantitative methods limits understanding of participants’ subjective thoughts, attitudes, and emotional responses, underscoring the need for nuanced interpretation within these constraints. In addition, as the data were collected through self-report measures, the findings may be subject to response biases such as social desirability. Furthermore, the cross-sectional design limits the ability to determine the directionality of relationships, and reverse causality cannot be ruled out. Finally, socioeconomic status was measured using a limited set of indicators, which may not fully capture its multidimensional structure. As the data were collected from students enrolled in public universities in Türkiye, the results may be context-specific and should be generalized to other cultural and institutional settings with caution. Additionally, it should be acknowledged that all authors of this study are male, which may have influenced the interpretation of gender-related findings. This limitation highlights the importance of incorporating more diverse and gender-sensitive perspectives in future research. Moreover, the unequal gender distribution in the sample (a higher proportion of female participants than male participants) may have affected the robustness of gender-based comparisons and should be considered when interpreting moderation effects. Finally, as the data were collected from students enrolled in public universities in Türkiye, the findings may be context-specific and should be generalized to other cultural and institutional settings with caution.

## Conclusion

The findings of this study underscore the significant relationships among school satisfaction, perceived future employability, socioeconomic status, and life satisfaction. Higher levels of school satisfaction were associated with increased perceived future employability, while both school satisfaction and perceived employability were positively related to life satisfaction. Perceived future employability emerged as a key mediating factor linking educational experiences to overall well-being, indicating that satisfaction with one’s educational experience shapes expectations about future career opportunities, which in turn influence life satisfaction. In addition, socioeconomic status was found to significantly affect both perceived future employability and life satisfaction, while gender moderated the relationships between school satisfaction, socioeconomic status, and perceived employability.

Beyond these empirical findings, the results suggest that individuals’ perceptions of their future are not solely determined by individual competencies but are also shaped by broader social structures, as emphasized in Pierre Bourdieu’s theoretical framework. In this respect, the study provides a theoretical contribution by demonstrating how Bourdieu’s concepts of capital and habitus can help explain the socially embedded nature of perceived future employability. Overall, this study contributes to the literature by integrating psychological and sociological perspectives and providing a more comprehensive understanding of how educational experiences and structural conditions jointly shape individuals’ perceptions and well-being outcomes.

From a practical perspective, the findings suggest that universities should prioritize student satisfaction and provide supportive educational environments, career guidance, and skill development opportunities to strengthen students’ perceptions of employability and overall well-being. In addition, policies aimed at reducing socioeconomic inequalities in education and access to resources may contribute to more equitable perceptions of future opportunities. Future research should employ longitudinal designs to better understand the directionality of these relationships and explore cross-cultural samples to examine the generalizability of the findings across different contexts.

### Implications

The research findings carry significant implications for educational institutions and policymakers. Prioritizing a supportive, engaging educational environment can influence students’ perceptions of future employability and overall life satisfaction. Recognizing the influence of school satisfaction on future career perceptions underscores the need for institutions to focus not only on academic success but also on nurturing skills and experiences valued in the job market. Additionally, the study highlights the importance of socioeconomic status in shaping perceptions of employability, urging policymakers to address disparities in access to education and opportunities. Gender differences in the relationship between satisfaction, socioeconomic status, and employability emphasize the need for targeted interventions that acknowledge and mitigate gender-based disparities in education and employment.

## Data Availability

The raw data supporting the conclusions of this article will be made available by the authors, without undue reservation.
